# Light-induced expression of gRNA allows for optogenetic gene editing of T lymphocytes *in vivo*

**DOI:** 10.1093/nar/gkaf213

**Published:** 2025-03-20

**Authors:** Diego Velasquez Pulgarin, Nathalie Pelo, Lin Ferrandiz, Tilen Tršelič, William A Nyberg, Gary Bowlin, Alexander Espinosa

**Affiliations:** Center for Molecular Medicine, Department of Medicine (K2), Karolinska Institutet, Karolinska University Hospital, 171 64 Stockholm, Sweden; Department of Hematology, St Jude Children’s Research Hospital, Memphis, TN 38105, United States; Center for Molecular Medicine, Department of Medicine (K2), Karolinska Institutet, Karolinska University Hospital, 171 64 Stockholm, Sweden; Center for Molecular Medicine, Department of Medicine (K2), Karolinska Institutet, Karolinska University Hospital, 171 64 Stockholm, Sweden; Center for Molecular Medicine, Department of Medicine (K2), Karolinska Institutet, Karolinska University Hospital, 171 64 Stockholm, Sweden; Department of Medicine (H7), Karolinska Institutet, Karolinska University Hospital, 141 83 Huddinge, Sweden; Department of Medicine, University of California, San Francisco, San Francisco, CA 94143, United States; Department of Biomedical Engineering, University of Memphis, Memphis, TN 38152, United States; Center for Molecular Medicine, Department of Medicine (K2), Karolinska Institutet, Karolinska University Hospital, 171 64 Stockholm, Sweden

## Abstract

There is currently a lack of tools capable of perturbing genes in both a precise and a spatiotemporal fashion. The flexibility of CRISPR (clustered regularly interspaced short palindromic repeats), coupled with light’s unparalleled spatiotemporal resolution deliverable from a controllable source, makes optogenetic CRISPR a well-suited solution for precise spatiotemporal gene perturbations. Here, we present a new optogenetic CRISPR tool (Blue Light-inducible Universal VPR-Improved Production of RGRs, BLU-VIPR) that diverges from prevailing split-Cas design strategies and instead focuses on optogenetic regulation of guide RNA (gRNA) production. We engineered BLU-VIPR around a new potent blue-light activated transcription factor (VPR-EL222) and ribozyme-flanked gRNA. The BLU-VIPR design is genetically encoded and ensures precise excision of multiple gRNAs from a single messenger RNA transcript. This simplified spatiotemporal gene perturbation and allowed for several types of optogenetic CRISPR, including indels, CRISPRa, and base editing. BLU-VIPR also worked *in vivo* with cells previously intractable to optogenetic gene editing, achieving optogenetic gene editing in T lymphocytes *in vivo*.

## Graphical Abstract

**Graphical Abstract:**
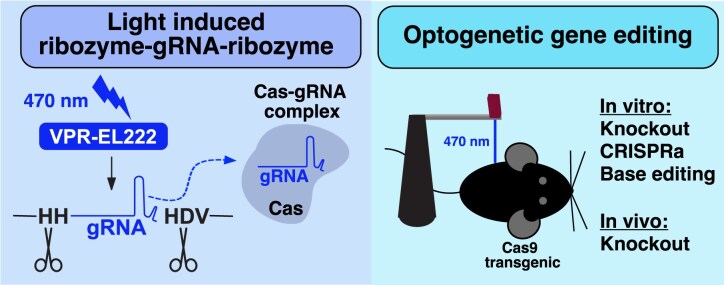

## Introduction

Clustered regularly interspaced short palindromic repeats (CRISPR) and their associated RNA-guided nucleases (Cas) have revolutionized genome engineering, and new systems and functionalities are being developed at a rapid pace. However, to harness the full power of CRISPR, it will be invaluable to establish spatiotemporal control of CRISPR systems *in vivo*. Since optogenetics is ideally suited for precise spatiotemporal control, optogenetic CRISPR has emerged as a promising method for spatiotemporal gene editing. Most current optogenetic CRISPR systems are based on light-induced dimerization of split-Cas [1], dimerization of Cas with effectors [1–5], single-chain modified Cas proteins [6, 7], or dissociation of anti-CRISPR proteins [8]. Although powerful, these systems have several disadvantages, including the requirement for cumbersome re-engineering to expand their use to additional Cas types and effectors, incompatibility with readily available Cas transgenic animal models, and their large sizes preventing delivery to primary cells using viral transduction. Alternatively, photocaged RNA, and hybrid genetic/chemical approaches, can be used for optogenetic CRISPR, but their limited half-life and short persistence *in vivo* reduce their usefulness [6, 9, 10]. To circumvent these limitations, we have generated a new optogenetic platform, Blue Light-inducible Universal VPR-Improved Production of RGRs (BLU-VIPR), allowing for simultaneous light-induced expression of genetically encoded single-guide RNAs (sgRNA) and proteins. BLU-VIPR can be combined with multiple Cas types and effectors and be delivered by virus for optogenetic gene editing *in vivo*. The flexibility and compatibility with existing Cas transgenic animal models make BLU-VIPR a novel optogenetic gene editing tool.

## Materials and methods

### Animals

We used the following mouse strains: Cas9 (B6J.129(Cg)-Gt(ROSA)26Sor^tm1.1(CAG-cas9*,-EGFP)Fezh^/J), TCRβ^−/−^ (B6.129P2-*Tcrb*^tm1Mom/J^), and CD45.1 (B6.SJL-Ptprc^a^ Pepc^b^/BoyJ). All mice were housed in a specific pathogen-free animal facility at Center for Molecular Medicine, Karolinska Institutet. The study was approved by the Ethical Review Committee North, Stockholm County (Ethical approval Dnr 7547-2022), and animals were handled in compliance with the guidelines at Karolinska Institutet.

### Cell lines and viruses

HEK293T cells (laboratory stock), Platinum-E cells (Cell Biolabs), and 293FT cells (Thermo Scientific) were cultured in high-glucose Dulbecco’s modified Eagle medium (DMEM) (Sigma–Aldrich) supplemented with fetal calf serum (10%) (Sigma–Aldrich), streptomycin (0.1 mg/ml) (Sigma–Aldrich), penicillin (100 U/ml), sodium pyruvate (1 mM) (Sigma–Aldrich), HEPES (10 mM) (Sigma–Aldrich), and l-glutamine (2 mM) (Sigma–Aldrich). Cells were routinely tested for mycoplasma contamination. Transfections of HEK293T and 293FT cells were performed using X-tremeGENE 9 DNA Transfection Reagent (Roche). Murine stem cell viruses (MSCVs) were packaged using Platinum-E cells by co-transfection of MSCV retroviral transfer plasmids with pCL-Eco (Addgene #12371) using X-tremeGENE 9 DNA Transfection Reagent (Roche). Lentiviruses were packaged using 293FT cells by co-transfection of lentiviral transfer plasmids with pMD2.G (Addgene #12259) and psPAX2 (Addgene #12260) using X-tremeGENE 9 DNA Transfection Reagent (Roche).

### Reporter cell lines

Reporter cell lines for Cas9 and base editors were generated using lentiviruses made from CRISPR-SP-Cas9 reporter (Addgene #62733), pLV-SI-121 (Addgene #131126), and pLV-SI-112 (Addgene #131127). HEK293T cells were transduced in six-well plates (2 × 10^5^ cells/well) with CRISPR-SP-Cas9 reporter, pLV-SI-121, or pLV-SI-112 lentivirus followed by selection with puromycin (2.5 μg/ml).

### Gene synthesis, Gibson assembly, and Golden Gate assembly

We synthesized short (0.2–1 kb) gene fragments as GeneStrands (Eurofins) or gBlocks (Integrated DNA Technologies). Longer (1–5 kb) gene fragments were generated by gene synthesis (Eurofins). Alternatively, gene fragments were amplified from plasmids by polymerase chain reaction (PCR) using Platinum Pfx DNA polymerase (Thermo Fisher). Constructs were assembled by Gibson assembly using the Gibson Assembly^®^ Master Mix (New England Biolabs) or by Golden Gate assembly. Assembled constructs were verified by Sanger sequencing and restriction digests. Other plasmids were obtained from commercial vendors or from the Addgene repository: pRL-CMV-Renilla (Promega), pRS0045 (Addgene #131124), pRS0035 (Addgene #131125), lentiCRISPR v2 (Addgene #52961), pLV-SI-121 (Addgene #131126), pLV-SI-112 (Addgene #131127), pCMV_ABEmax (Addgene #112095), pXR001 (Addgene #109049), pSBtet-GB (Addgene #60504), gRNA-Cloning vector (Addgene #41824), SP-dCas9-VPR (Addgene #63798), CRISPR-SP-Cas9 reporter (Addgene #62733), pCL-Eco (Addgene #12371), pMD2.G (Addgene #12259), and psPAX2 (Addgene #12260).

### RGR cloning into BLU-VIPR vectors

Ribozyme-gRNA-ribozymes (RGRs) were designed so that the first six nucleotides of the HH (hammerhead) ribozyme are the reverse complement of the first six nucleotides immediately following the HH ribozyme’s 3′ end. RGRs were cloned into BLU-VIPR vectors via Golden Gate assembly (SapI/LguI or BbsI). RGRs thus consist of flanking type IIS enzyme recognition sequences (with appropriate overhangs for directional insertion into each vector), an HH ribozyme, a gRNA, and an HDV (hepatitis delta virus) ribozyme. The sequences for all these elements are outlined in Supplementary Table S1. An Excel-based RGR generator is available to download allowing for easy generation of RGR sequences for a desired gRNA by the end user (https://www.addgene.org/220498/). Complete RGRs were ordered as double-stranded fragments and subsequently cloned into BLU-VIPR plasmids.

### BLU-VIPR kinetics

For ON kinetics, HEK293T cells were seeded in six-well plates (6.0 × 10^5^ per well), transfected with 500 ng BV plasmid using XtremeGene 9 (Roche). Twenty-four hours after transfection, cells were illuminated (1 mW/cm^2^, 20 s ON, 40 s OFF, 470 nm) for the indicated time points. After illumination, cells were kept in the dark for 3 h and prepared for analysis by flow cytometry. Briefly, the cells were resuspended in phosphate buffered saline (PBS) with two drops of NucBlue™ Fixed Cell ReadyProbes™ Reagent (Thermo Fisher) per ml, incubated for 20 min, and fixed using 4% paraformaldehyde (PFA) for 10 min at room temperature. For OFF kinetics, HEK293T cells were seeded in 12-well plates (1.5 × 10^5^ cells per well) and transfected with 150 ng BV plasmid using XtremeGene 9 according to the manufacturer’s protocol. Twenty-four hours after transfection, cells were illuminated (1 mW/cm^2^, 20 s ON, 40 s OFF, 470 nm) for 3 h. Cells were collected at indicated time points and RNA was extracted using TRIzol (Invitrogen) followed by digestion of DNA with amplification grade DNase I (Thermo Fisher). mCherry transcript quantification was performed using quantitative reverse transcription PCR (RT-qPCR).

### Transilluminators

Two blue light-emitting diode (LED) setups were designed and built for delivery of blue light to cells in culture. The first consists of a commercially available transilluminator (large blue LED transilluminator, IO Rodeo), controlled by an Arduino microcontroller (UNO, Arduino) and a relay module (SRD-05VDC-SL-C, Songle). The transilluminator was mounted inside a cell culture incubator. The second setup was custom-made for experiments with black-walled, optical bottom 96-well plates. It consists of individually controlled LEDs (one LED per well), driven by LED drivers (STP16CP05, STM Microelectronics), directed by a TEENSY 3.2 microcontroller (PJRC). The LED array is housed in a standoff-supported, laser-cut ABS enclosure, aligning each LED to a determinate well. This system allows for individual control of light intensity and duration on a well-per-well basis. Both setups were calibrated for light intensity using a custom-built photodiode (PDB-C139, Advanced Photonics) Arduino pyranometer.

### Guide RNAs and primers

The gRNAs for Cas9 reporter, base editing reporters, *IL1RN*,*HBG1*/*HBG2*,*NEAT1*, and *Thy1* were selected based on published gRNA sequences [3, 11, 12]. The nontargeting control (NTC) gRNA was selected based on published gRNA sequences [13]. To identify potent gRNAs for CRISPRa, we screened at least eight gRNAs for *BMP2* and *PDGFB* (not shown). The gRNA sequences used for the *BMP2* and *PDGFB* gRNAs were designed with the Genetic Perturbation Platform’s sgRNA Designer web tool (Broad Institute). gRNA sequences were chosen to target 75–300 base pairs upstream of the transcription start site using the human GRCh38 reference genome and to be used with SP-Cas9 (NGG PAM) or LB-Cas12a (TTTV PAM). All gRNAs and primers used in this study are listed in Supplementary Tables S2 and S3.

### Quantitative RT-PCR

All RNA extractions were made using TRIzol (Invitrogen), and conversion of RNA to complementary DNA (cDNA) was performed using the High-Capacity cDNA Reverse Transcription Kit (Applied Biosystems). For gene expression analysis, we performed RT-qPCR using TaqMan™ Universal Master Mix II, no UNG (Thermo Fisher Scientific), with the following TaqMan probes: *HPRT1* (Hs01003267_m1), *BMP2* (Hs00154192_m1), mCherry (Mr07319439_mr), and *PDGFB* (Hs00966522_m1). SYBR Green primers for RT-qPCR expression analysis for IL1RN and HBG1/2 are listed in Supplementary Table S3. A LightCycler 96 real-time PCR system (Roche) was used for real-time PCR, and data were analyzed using the LightCycler 1.1 software (Roche).

### Optogenetic knockouts and CRISPRa using BLU-VIPR

For the BLU-VIPR activity assays, 4 × 10^4^ cells HEK293T cells were seeded in black-walled, optical bottom 96-well plates. Cells were transfected with 50 ng BLU-VIPR plasmid (Addgene #220498) immediately after seeding. Twenty-four hours post-transfection, cells were exposed to 1 mW/cm^2^ of 470 nm light (pulsed, 20 s ON, 60 s OFF) on a custom-made blue LED transilluminator for 24 h. After light exposure, fluorescence micrographs were taken on a ZOE fluorescent cell imager (Bio-Rad). To measure optogenetic activation of Cas9, 4 × 10^4^ Cas9 reporter cells were seeded in black-walled, optical bottom 96-well plates and transfected with 30 ng lentiCRISPRv2 (without gRNA) and 50 ng BLU-VIPR plasmids with targeting and nontargeting gRNAs. Twenty-four hours post-transfection, cells were exposed to pulses (20 s ON, 60 s OFF) of 1 mW/cm^2^ of 470 nm light or kept in the dark. After 48 h of light exposure, fluorescence micrographs were taken on a ZOE Fluorescent Cell Imager (Bio-Rad). For CRISPRa assays, 4 × 10^4^ HEK293T cells were seeded in black-walled, optical bottom 96-well plates and transfected with 50 ng SP-dCas9-VPR, or 3 ng LB-dCas12a-VPR, and 50 ng BLU-VIPR plasmids with targeting gRNAs. Twenty-four hours post-transfection, cells were exposed to pulses (20 s ON, 60 s OFF) of 1 mW/cm^2^ of 470 nm light, or kept in the dark, and harvested 24 h later for RNA extraction and RT-qPCR. Illumination regimes for these experiments were determined based on maximizing light-induced RGR production, convenience, and downstream activity of gRNA and Cas effectors.

### Optogenetic base editing using BLU-VIPR

Base editor reporter cells (4 × 10^4^ cells) were co-transfected with 50 ng pCMV_ABEmax or pRS0035 (Target-AID) with 50 ng of BLU-VIPR plasmids containing targeting or nontargeting gRNAs. After 24 h, cells were exposed to pulses (20 s ON, 60 s OFF) of 1 mW/cm^2^ of 470 nm light or kept in the dark. For flow cytometric analysis of the EGFP (enhanced green fluorescent protein) reporter, cells were harvested 24 h after light exposure and analyzed using a Gallios Flow Cytometer (Beckman Coulter). For imaging of the EGFP reporter, base editor cells were kept in the dark for 48 h after light exposure, and fluorescence micrographs were taken on a ZOE Fluorescent Cell Imager (Bio-Rad). To achieve optogenetic base editing of an endogenous gene, 4 × 10^4^ 293FT cells were seeded in black-walled, optical bottom 96-well plates and transfected with 50 ng pRS0035 and 50 ng BLU-VIPR plasmids with *NEAT1* targeting or nontargeting gRNA. Twenty-four hours post-transfection, cells were exposed to pulses (20 s ON, 60 s OFF) of 1 mW/cm^2^ of 470 nm light for 48 h or kept in the dark. Light-exposed cells were harvested and sorted for mCherry expression on a Sony SH800 cell sorter (Sony Biotechnology). Genomic DNA was extracted from cells exposed to light, and from cells kept in the dark, using the Monarch Genomic DNA Purification Kit following the manufacturer’s protocol (New England Biolabs). The targeted region in *NEAT1* was amplified by genomic PCR (primers in Supplementary Table S4). Amplicons were first sequenced by Sanger sequencing and analyzed with EditR software [14]. Next, to obtain precise quantification of edits, we performed Illumina sequencing. Briefly, a sequencing library was generated by equimolar pooling of indexed genomic PCR products of endogenous *NEAT1*. The PCR products were isolated using QIAquick Gel Extraction Kit (Qiagen) and quality control was performed with the Agilent High Sensitivity D1000 ScreenTape System (Agilent Technologies). The pooled library was sequenced using the MiSeq System with the MiSeq Micro Flow Cell (4 million reads) paired-end i7 indexed reads (150 cycles). Fastq files were demultiplexed and analyzed with CRISPResso2 for base editing (GEO accession number GSE275757). Illumination regimes for these experiments were determined based on maximizing light-induced RGR production, convenience, and downstream activity of gRNA and Cas effectors.

### Retroviral transduction

Mouse T lymphocytes were isolated from Cas9 transgenic mice and transduced following a modified protocol by Kurachi *et al.* [12]. Briefly, splenic T lymphocytes were isolated from Cas9 transgenic mice using negative selection with the EasySep Mouse T Cell Isolation Kit (StemCell Technologies) and then activated with Dynabeads Mouse T-Activator CD3/CD28 beads (Cat# 11456D, Thermo Scientific). The isolated T lymphocytes were cultured in optogenetic RPMI 1640 medium (Cat# 11835030, Thermo Scientific), lacking phenol red and HEPES, supplemented with 200 U/ml recombinant human IL-2 (Peprotech). One day after activation, the T lymphocytes were transduced with MSCV-BLU-VIPR retrovirus by spinfection (2000 rcf, 60 min). MSCV-BLU-VIPR retroviruses, containing Thy1.2-specific gRNA or NTC gRNA, were packaged by transfecting Platinum-E cells with pCL-Eco and MSCV-BLU-VIPR plasmids (Addgene #220497) containing Thy1.2-specific gRNA or NTC gRNA.

### Optogenetic gene editing in primary mouse T lymphocytes

For optogenetic CRISPR in primary mouse T lymphocytes *in vitro*, Cas9^+^ T lymphocytes were transduced with MSCV-BLU-VIPR containing Thy1.2-specific or NTC gRNA in six-well plates, and one day later the transduced cells were transferred to black-walled, optical bottom 96-well plates. T lymphocytes were then exposed for 48 h to pulses of 1 mW/cm^2^ 470 nm light (20 s ON, 60 s OFF) or kept in dark conditions. Following this exposure period, all cells were kept in the dark for 72 h and then stained for Thy1.2. The expression of Thy1.2 was analyzed using flow cytometry. For optogenetic CRISPR *in vivo*, Cas9^+^ CD45.1^+^ T lymphocytes were transduced with MSCV-BLU-VIPR containing Thy1.2-specific or NTC gRNA and one day later injected intravenously (5 × 10^6^ cells) into CD45.2^+^ TCRβ^−/−^ mice. Following 3–8 weeks of homeostatic T cell expansion, the recipients were anesthetized (isoflurane 2%) and the mice were prepared for intravital optogenetic stimulation following a protocol adapted from Ulrich von Andrian [15]. Briefly, the skin with the left inguinal lymph node (iLN) was flipped inside out following a small incision immediately left to the midline and single suture traction was established to keep the LN exposed. The tissue was kept moist with isotonic saline (0.9% NaCl). A stimulation chamber was built around the lymph node using vacuum grease to prevent the isotonic saline from leaking out. The fiber optic cannula (Thorlabs) was placed directly over the lymph node for 1 h of light stimulation (1 mW/cm^2^, 20 s ON, 40 s OFF, 470 nm). For analysis of optogenetic CRISPR experiments, secondary lymphoid organs and blood were collected 48 h post-illumination and Thy1.2 expression was analyzed by flow cytometry. Illumination regimes for these *in vivo* experiments were determined based on desired light-induced RGR production, surgical limitations, and downstream activity of gRNA and Cas effectors.

### Flow cytometry

Dead cells were excluded by incubating with the dead cell stain SYTOX™ Red (Invitrogen) before acquisition according to the manufacturer’s protocol. To generate monostain controls for compensation, we used Ultracomp Plus Beads (eBioscience), GFP BrightComp eBeads™ Compensation Bead Kit (Thermo Scientific), and mCherry Flow Cytometer Calibration Beads (Takara). Flow cytometry of stained T lymphocytes was then performed using a BD LSRFortessa Cell Analyzer (BD Biosciences) and the data were analyzed using FlowJo v10 (BD). The following antibodies were used for flow cytometry: anti-mouse Thy1.2 Brilliant Violet 785 (clone 30-H12, BioLegend), anti-mouse Thy1.1 Brilliant Violet 421 (clone OX-7, BioLegend), anti-mouse CD45.1 PE/Dazzle 594 (clone A20, BioLegend), anti-mouse CD45.2-Alexa Fluor 532 [clone 104, Invitrogen (eBioscience)], and anti-mouse CD3ϵ APC (clone 145-2C11, BD Pharmingen).

## Results

### Simultaneous induction of protein and gRNA from a blue-light induced Pol II promoter

**Figure 1.**
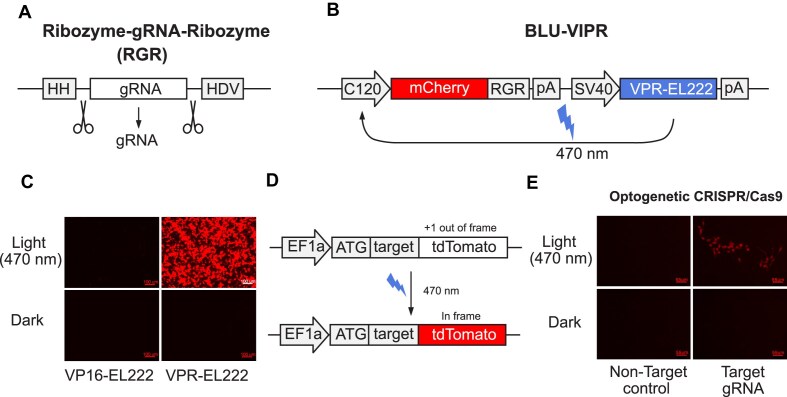
BLU-VIPR allows for optogenetic CRISPR using light-induced expression of gRNA. (**A**) The RGR design consists of an HH ribozyme followed by the gRNA and an HDV ribozyme. After precise self-cleavage of the ribozymes, a functional gRNA is released. (**B**) Design of construct for VPR-EL222-dependent activation of C120 promoter transcription, allowing for simultaneous expression of mCherry and gRNA after exposure to blue light (470 nm). (**C**) Comparison of mCherry reporter expression in HEK293T cells after transfection with VPR-EL222 or VP16-EL222 constructs followed by exposure to blue light for 24 h. The experiment was performed three times and one representative image is shown. Scale bars are 100 μm. (**D**) Out-of-frame Cas9 reporter where the expression of tdTomato is restored after Cas9-mediated indels. (**E**) HEK293T cells were transfected with BLU-VIPR (without mCherry) containing a gRNA targeting the out-of-frame sequence in tdTomato and Cas9. After 48 h of light exposure, the cells were cultured for an additional 72 h before detection of tdTomato demonstrating successful optogenetic induction of indels by Cas9. Scale bars are 55 μm. The experiment was performed three times and one representative image is shown.

To enable light-induced expression of gRNAs, we took advantage of the blue-light activated protein EL222 [16]. Upon exposure to blue light (470 nm), EL222 reversibly homodimerizes and binds to its response elements in the C120 promoter [16]. By fusing EL222 to a transcriptional activator, it is therefore possible to activate RNA polymerase II (RNAPII)-mediated transcription from the C120 promoter by exposure to blue light (Supplementary Fig. S1A). To ensure strong expression of gRNA after light exposure, we engineered a new potent blue-light induced transcription factor (VPR-EL222) by fusing EL222 to the synthetic transcriptional activator VP64-p65-Rta (VPR) [17]. To enable the release of a functional gRNA from the resulting transcript, we flanked the gRNA with self-cleaving HH and HDV ribozymes in an RGR design [18–20] (Fig. 1A). These ribozymes precisely excise the gRNA from the transcript and allow for simultaneous production of functional gRNAs and proteins from the same transcript. We then generated a VPR-EL222 expressing construct with both RGR and an mCherry reporter under the control of the C120 promoter (Fig. 1B). To test the construct, we used LED transilluminators for controllable delivery of blue light (470 nm) to transfected HEK293T cells. Indeed, VPR-EL222 induced high levels of mCherry only after exposure to blue light, with no observable leakiness when kept in dark conditions, in a reversible and re-activatable manner (Fig. 1C and Supplementary Figs S1B and S6). In contrast, a fusion of EL222 to the commonly used transcriptional activator VP16 (VP16-EL222) was unable to induce mCherry (Fig. 1C). This lack of robust light-induced expression by VP16-EL222 has been confirmed by Gabel *et al.*[21]. The robust VPR-EL222-based system for light-induced expression of gRNA was designated BLU-VIPR (Supplementary Fig. S2A and B) (available from Addgene #220498).

To establish whether BLU-VIPR could be used for optogenetic induction of Cas9-mediated double-stranded DNA breaks, we generated Cas9 reporter cells containing an out-of-frame (+1 bp) tdTomato under control of the EF1α promoter [22] (Fig. 1D). Cas9 reporter cells were then co-transfected with a Cas9 plasmid and a BLU-VIPR plasmid containing RGRs with a gRNA targeting the frameshifted sequence of tdTomato, or a nontargeting control gRNA (we removed mCherry from the BLU-VIPR construct to avoid interference with tdTomato). Indeed, we could verify that exposure to blue light resulted in tdTomato fluorescence, indicating reading-frame restoration and successful optogenetic control of Cas9 (Fig. 1E). In summary, we engineered a system, BLU-VIPR, for potent induction of functional gRNA and protein from the same transcript upon exposure to blue light.

### Multiplexed and orthogonal optogenetic CRISPR activation

**Figure 2.**
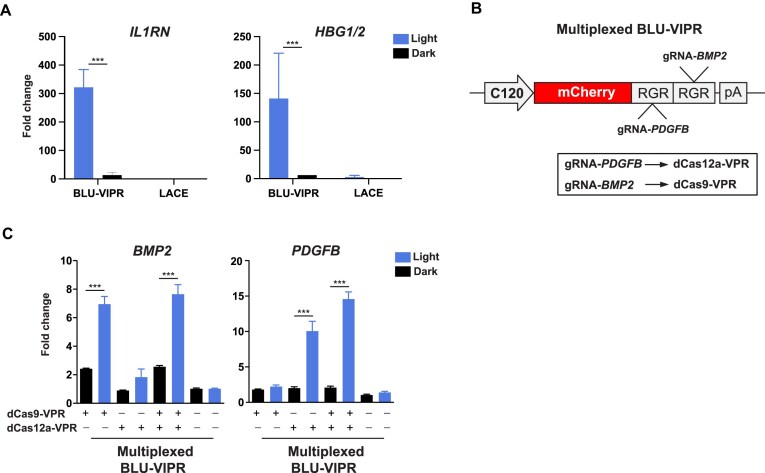
BLU-VIPR allows for multiplexed and orthogonal optogenetic CRISPRa. (**A**) BLU-VIPR activation of endogenous genes, compared to LACE. BLU-VIPR achieved robust activation of target genes upon stimulation with light using only one gRNA per target, while LACE failed to activate transcription with one gRNA in HEK293T cells. (**B**) RGR design for multiplexed activation of dCas12a-VPR and dCas9-VPR. (**C**) Multiplexed, orthogonal optogenetic dCas12a-VPR and Cas9-VPR to activate the endogenous genes *PDGFB* and *BMP2*. HEK293T cells were transfected with BLU-VIPR-multiplexed, and either dCas12a-VPR or dCas9-VPR, or both. Gene expression was assayed after 24 h of light stimulation, normalized to *HPRT1* expression, and fold change is relative to untransfected samples kept under dark conditions. The experiment was performed with *n* = 5. One-way ANOVA with Tukey post-hoc test was used to identify statistical significance (***P< .001).

To test whether BLU-VIPR could robustly activate gene expression with blue light stimulation, we first compared BLU-VIPR to LACE, a previous optogenetic CRISPR activation (CRISPRa) system based on optogenetic recruitment of functional domains to dCas9 [3]. In contrast to LACE, which requires four gRNAs to effectively induce gene expression [3, 23], BLU-VIPR was able to strongly induce *IL1RN*, *HBG1/2*, and *BMP2* with a single gRNA after light exposure (Fig. 2A and Supplementary Fig. S2C). Furthermore, BLU-VIPR (using a single gRNA) achieves comparable activation to LACE (using four gRNAs) [3]. Since the RGR design of BLU-VIPR allows for the expression of multiple gRNAs from the same light-induced RNA transcript, we reasoned that it should enable multiplexed and orthogonal optogenetic CRISPR. To demonstrate this, we used the BLU-VIPR system for multiplexed and orthogonal CRISPRa based on dCas9-VPR and dCas12a-VPR. We achieved this by inserting two RGRs into the BLU-VIPR construct. The first RGR contained a gRNA for dCas12a-VPR-dependent activation of *PDGFB* (platelet-derived growth factor subunit B), and the second RGR contained a gRNA for dCas9-VPR-dependent activation of *BMP2* (bone morphogenetic protein 2) (Fig. 2B). This multiplexed RGR design thus allowed for simultaneous expression of two gRNAs from the same transcript. We then co-transfected the multiplexed BLU-VIPR construct together with dCas12a-VPR and dCas9-VPR and measured the induction of *PDGFB* and *BMP2* expression after exposure to blue light. Indeed, the multiplexed BLU-VIPR was able to achieve robust optogenetic activation of both *PDGFB* and *BMP2* expression (Fig. 2C). Importantly, the multiplexed RGR design was truly orthogonal since the *PDFGB*-specific gRNA was unable to activate *PDFGB* transcription with dCas9-VPR, and conversely, the *BMP2*-specific gRNA was unable to activate *BMP2* transcription with dCas12a-VPR. Altogether, these results demonstrate that BLU-VIPR is a VPR-EL222-based gRNA expression system that allows for robust optogenetic CRISPRa, and multiplexed and orthogonal optogenetic activation of dCas9-VPR and dCas12a-VPR. The reversible nature of EL222 light-induced dimerization and BLU-VIPR RGR production (Supplementary Fig. S6) suggests that reversible applications, such as CRISPRa, would be controllable in duration, and/or reactivation, by the illumination regime chosen for the experiment. The variables involved in CRISPRa modulation of endogenous genes would necessitate characterization of the kinetics for each target/guide/Cas combination to allow accurate temporal regulation.

### Optogenetic C-to-T and A-to-G base editing

**Figure 3.**
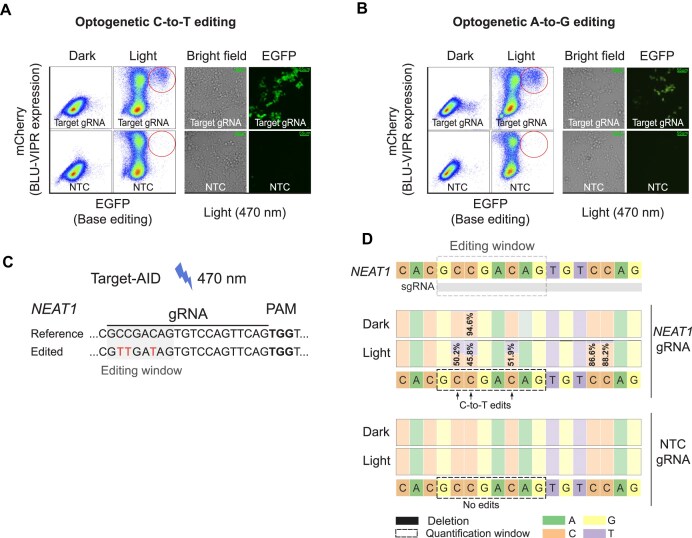
BLU-VIPR allows for optogenetic C-to-T and A-to-G base editing. (**A**) C-to-T reporter HEK293T cells, expressing EGFP upon successful C-to-T base editing, were transfected with Target-AID and BLU-VIPR containing a gRNA specific for the relevant target sequence in EGFP, or a nontargeting gRNA. Reporter cells were then illuminated, or kept under dark conditions, for 48 h and analyzed by flow cytometry. Detection of mCherry^+^ EGFP^+^ cells is evidence of successful optogenetic C-to-T base editing. Fluorescence microscopy confirmed the presence of EGFP^+^ cells after optogenetic C-to-T base editing in reporter cells. The experiment was repeated three times and one representative result is shown. Scale bars are 55 μm. (**B**) A-to-G reporter HEK293T cells, expressing EGFP upon successful A-to-G base editing, were transfected with ABEmax and BLU-VIPR containing a gRNA specific for the relevant target sequence in EGFP, or a nontargeting gRNA. Reporter cells were then illuminated, or kept under dark conditions, for 48 h and analyzed by flow cytometry. Detection of mCherry^+^ EGFP^+^ cells is evidence of successful optogenetic A-to-G base editing. The experiment was repeated three times and one representative result is shown. Scale bars are 55 μm. (**C**) Design of gRNA for optogenetic C-to-T base editing of endogenous *NEAT1*. (**D**) To precisely determine the frequency of C-to-T edits in *NEAT1* after exposure to light, we performed Illumina sequencing of *NEAT1* containing PCR products from sorted mCherry positive cells. Numbers represent the percentages of unedited reads. Representative data from two experiments are shown.

The ability to achieve optogenetic control of both Cas9 and Cas12 demonstrated that BLU-VIPR is compatible with different types of Cas proteins. We therefore asked whether BLU-VIPR also could achieve control of Cas variants previously intractable for optogenetic CRISPR. To answer this, we tested whether BLU-VIPR could induce C-to-T and A-to-G base editing after light exposure. To test this, we first generated C-to-T and A-to-G base editing reporter cells (Supplementary Fig. S3A and B), and then co-transfected them with Target-AID (C-to-T base editor) [11], or ABEmax (A-to-G base editor) [24], together with BLU-VIPR plasmids containing gRNAs specific for the target sites in the respective reporters. After exposure to blue light, analysis by flow cytometry revealed populations of mCherry^+^ EGFP^+^ cells, indicating activation of BLU-VIPR (mCherry^+^) and successful optogenetic C-to-T or A-to-G base editing (EGFP^+^) (Fig. 3A and B). To test whether the BLU-VIPR system also was capable of optogenetic base editing of endogenous genes, we transfected 293FT cells with Target-AID and BLU-VIPR containing a *NEAT1* (nuclear enriched abundant transcript 1)-specific gRNA (Fig. 3C). After exposure to blue light, we sorted mCherry^+^ cells by flow cytometry and sequenced the targeted region in *NEAT1* (Fig. 3D). We could demonstrate successful optogenetic C-to-T base editing in the editing window (between 48% and 54%) using Illumina sequencing. This level of editing matches reported editing efficiency of Target-AID [25], thus indicating that the performance of the base editor is the limiting factor in this experiment. In summary, we demonstrated successful optogenetic induction of C-to-T and A-to-G base editing, including the successful optogenetic C-to-T base editing of an endogenous gene. To our knowledge, this is one of the first demonstrations of successful optogenetic base editing of an endogenous gene.

### Optogenetic gene editing in primary T lymphocytes

**Figure 4.**
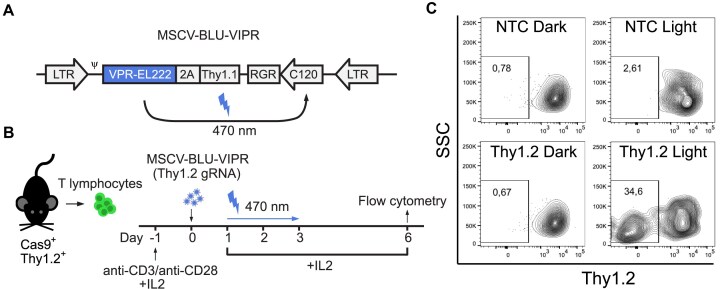
Optogenetic gene editing of primary mouse Cas9^+^ T lymphocytes *in vitro*. (**A**) Design of the retroviral MSCV-BLU-VIPR constructs for blue-light induced expression of gRNA after transduction of primary mouse T lymphocytes. (**B**) Cas9^+^ splenic mouse T lymphocytes (Thy1.2^+^) were transduced with MSCV-BLU-VIPR containing Thy1.2-specific or NTC gRNA, followed by exposure to blue light and analysis by flow cytometry. (**C**) After 48 h of light exposure, followed by a 72-h dark period, the T lymphocytes were stained for Thy1.2, gated for singlets and viability, and then analyzed for Thy1.2 expression. Representative result from two experiments is shown.

Optogenetic gene editing in hard-to-transfect primary cells is challenging due to the necessity of delivering large Cas-based optogenetic machineries into cells [9]. In contrast, the small size of the BLU-VIPR system allows it to be introduced to primary cells using efficient viral delivery. This ease of delivery, coupled with wide availability of Cas transgenic animal models, allows for expedient and facile development of flexible optogenetic models using BLU-VIPR. To demonstrate that BLU-VIPR can be used for optogenetic CRISPR gene editing in primary cells, we transduced Cas9 transgenic (Cas9^+^) primary mouse T lymphocytes with a retrovirus containing BLU-VIPR. First, we generated MSCV constructs for retroviral delivery of BLU-VIPR (MSCV-BLU-VIPR) (available from Addgene #220497) containing a gRNA specific for *Thy1* (Thy1.2) or an NTC gRNA (Fig. 4A). We then isolated splenic T lymphocytes from Cas9 transgenic mice [12], followed by transduction with MSCV-BLU-VIPR retrovirus and exposure to pulsed blue light for 48 h (Fig. 4B).

After light exposure, the T lymphocytes were left in the dark for 72 h before analysis of Thy1.2 expression by flow cytometry (gating strategy in Supplementary Fig. S4A). Indeed, Thy1.2 knockout T lymphocytes were only detected after transduction with Thy1.2-specific MSCV-BLU-VIPR and exposure to blue light (Fig. 4C). We did not detect loss of Thy1.2 either in T lymphocytes that were transduced with Thy1.2-specific MSCV-BLU-VIPR virus and kept in the dark or in T lymphocytes transduced with NTC control MSCV-BLU-VIPR virus and exposed to blue light. Altogether, these results demonstrate that BLU-VIPR enables optogenetic gene editing in primary T lymphocytes. To our knowledge, this makes BLU-VIPR the first optogenetic CRISPR system to allow for optogenetic gene editing in primary T lymphocytes.

### Optogenetic gene editing of T lymphocytes in lymph nodes

**Figure 5.**
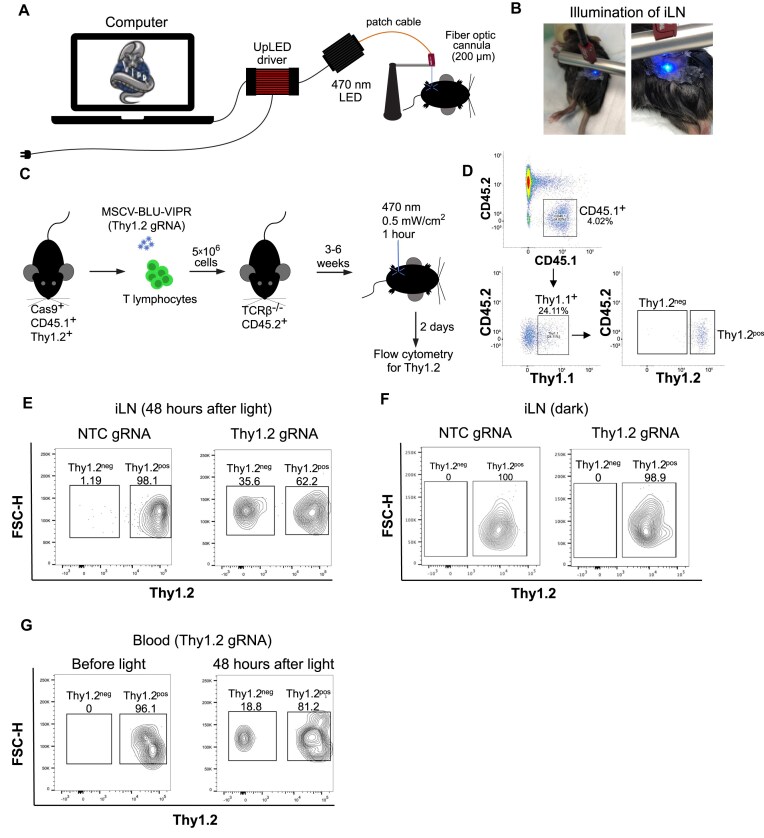
BLU-VIPR allows for optogenetic gene editing of Cas9^+^ T lymphocytes *in vivo*. (**A**) Optogenetic setup for illumination of lymph nodes with blue light (470 nm). (**B**) Illumination of a single iLN. (**C**) Cas9^+^ splenic mouse T lymphocytes (CD45.1^+^ Thy1.2^+^) were transduced with MSCV-BLU-VIPR containing Thy1.2-specific or NTC gRNA. The transduced cells were adoptively transferred to TCRβ^−/−^ CD45.2^+^ mice. After reconstitution of the T cell lymphocyte pool, we illuminated an iLN with blue light to induce gene editing of Thy1.2. (**D**) Gating strategy to determine levels of Thy1.2 by flow cytometry. (**E**) The levels of Thy1.2 on transduced Cas9^+^ T lymphocytes (CD45.1^+^ Thy1.1^+^) from illuminated iLNs were determined by flow cytometry. (**F**) The levels of Thy1.2 on transduced Cas9^+^ T lymphocytes (CD45.1^+^ Thy1.1^+^) from nonilluminated mice were determined by flow cytometry. (**G**) The levels of Thy1.2 on transduced Cas9^+^ T lymphocytes (CD45.1^+^ Thy1.1^+^) in blood before and after illumination of an iLN were determined by flow cytometry.

Light-based gene perturbations of T lymphocytes *in vivo* would allow for spatiotemporal dissection of immune responses with unparalleled precision and pave the way for new insights into immune responses. This could, for example, be used to spatiotemporally dissect antitumoral immune responses to inform and improve immunotherapies against a variety of cancer types. To test whether the BLU-VIPR system could be harnessed for precise optogenetic CRISPR in T lymphocytes *in vivo*, we first built an optogenetic setup allowing for precise illumination of individual lymph nodes in mice (Fig. 5A and B). We then asked whether BLU-VIPR was tolerated by T lymphocytes *in vivo*, and to answer this, we transduced Cas9^+^ CD45.1^+^ T lymphocytes with MSCV-BLU-VIPR followed by adoptive transfer into CD45.2^+^ TCRβ^−/−^ mice. Indeed, Cas9^+^ T lymphocytes still expressed BLU-VIPR (Thy1.1^+^) after >20 weeks without loss of frequency or expression level, demonstrating that BLU-VIPR is tolerable to, and persists in, T lymphocytes *in vivo* (Supplementary Fig. S5). Next, we asked whether we could achieve optogenetic gene editing *in vivo*, and to answer this, we transduced Cas9^+^ CD45.1^+^ T lymphocytes with MSCV-BLU-VIPR retrovirus containing NTC or Thy1.2-specific gRNAs followed by adoptive transfer into CD45.2^+^ TCRβ^−/−^ mice. Between 3 and 6 weeks after adoptive transfer, we delivered pulsed blue light (1 mW/cm^2^, 20 s ON, 40 s OFF, 470 nm) to a single iLN to induce the expression of gRNA (Fig. 5C). After 1 h of light exposure, we sutured the surgical incision, and 48 h later, we used flow cytometry to analyze the expression of Thy1.2 in transduced Cas9^+^ T lymphocytes (CD45.1^+^ Thy1.1^+^) in secondary lymphoid organs and blood. To assess the efficiency of optogenetic gene editing, we gated on transferred Cas9^+^ T lymphocytes expressing BLU-VIPR (CD45.1^+^ Thy1.1^+^) and determined the frequencies of Thy1.2-positive (Thy1.2^pos^) and Thy1.2-negative (Thy1.2^neg^) cells (Fig. 5D). Indeed, 48 h after illumination of iLNs, we found that >35% of CD45.1^+^ Thy1.1^+^ T lymphocytes transduced with Thy1.2-specific MSCV-BLU-VIPR virus were Thy1.2^neg^, demonstrating successful optogenetic gene editing *in vivo* (Fig. 5E). In contrast, Thy1.2 levels were not lost in CD45.1^+^ Thy1.1^+^ T lymphocytes in mice without exposure of iLNs to blue light, demonstrating that the MSCV-BLU-VIPR system requires blue light to induce gene editing (Fig. 5F). Cas9^+^ T lymphocytes transduced with NTC control MSCV-BLU-VIPR virus did not lose expression of Thy1.2 after illumination (Fig. 5F). To further verify that the loss of Thy1.2 was induced by light illumination, we determined the expression of Thy1.2 on BLU-VIPR expressing Cas9^+^ T lymphocytes (CD45.1^+^ Thy1.1^+^) cells in blood before and 48 h after illumination of iLNs. Before illumination of iLNs, no Thy1.2^neg^ CD45.1^+^ Thy1.1^+^ T lymphocytes were detected in blood; in contrast, after illumination >18% CD45.1^+^ Thy1.1^+^ T lymphocytes were Thy1.1^neg^ (Fig. 5G). In all, these results demonstrate that BLU-VIPR can be used for optogenetic gene editing in T lymphocytes *in vivo*.

## Discussion

We have engineered a potent light-responsive transcription factor (VPR-EL222) that can trigger the simultaneous expression of proteins and gRNAs from an RNAPII promoter. The ribozyme-flanked gRNA design ensures precise excision of multiple gRNAs from a single transcript, therefore also allowing for multiplexed optogenetic gene editing. This new multipurpose optogenetic CRISPR platform (BLU-VIPR) can be combined with multiple Cas types and effectors and is deliverable by virus. Therefore, BLU-VIPR provides unprecedented flexibility for optogenetic CRISPR gene editing, allowing for efficient optogenetic knockouts in Cas9 transgenic primary T lymphocytes *in vivo*. The ability of BLU-VIPR to be introduced to primary cells using viral delivery systems confers several advantages, including its use in a wide range of cell types and its stable expression after integration in the genome. While the BLU-VIPR system is applicable to various cell types, tissues, and animal models, we chose to test the *in vivo* optogenetic gene editing in mouse T lymphocytes due to the clinical relevance of therapeutic T lymphocytes. Furthermore, since therapeutic T lymphocytes [e.g. tumor-infiltrating lymphocytes and chimeric antigen receptor (CAR) T lymphocytes] are generated *ex vivo*, they could potentially be modified with BLU-VIPR before adoptive transfer to tumor-bearing recipients in research models. Emerging viral delivery technologies using directed evolution-derived adeno-associated virus (AAV) with high tropism for murine T lymphocytes would circumvent *ex vivo* engineering and even target tissue-resident T lymphocytes [26, 27]. This facile generation of optogenetic CRISPR murine T lymphocyte models could, therefore, be a useful tool for the precise spatiotemporal control of anti-tumor responses to better understand biological phenomena and ultimately develop technologies to improve tumor killing and reduce toxicities, in addition to allowing for the exploration of cancer immunotherapy questions requiring refined control of the genome in time and space.

While optogenetic approaches are well suited for spatiotemporal perturbations, a CRISPR/Cas-based optogenetic system is inherently tethered to the processes associated with gene editing by Cas proteins. This reliance on a cascade of events post-illumination limits the temporal resolution of the BLU-VIPR system, and the exact requirements for each experiment must be therefore carefully considered. Furthermore, while BLU-VIPR allows for unprecedented flexibility, as a gRNA-centered optogenetic platform, it is reliant on Cas being present in the target cell; however, this can easily be achieved by co-delivery of Cas or the use of Cas transgenic animal models. Likewise, the *in vivo* optogenetic model presented in this paper depends on homeostatic proliferation to reconstitute the T lymphocyte population in TCRβ knockout recipients. While reliance on homeostatic proliferation can be a drawback when investigating immune responses *in vivo*, adoptive transfer of MSCV-BLU-VIPR-transduced TCR transgenic or CAR T lymphocytes should circumvent this limitation [26, 28, 29]. Ultimately, BLU-VIPR enables the transformation of an off-the-shelf transgenic Cas model into an optogenetic gene perturbation-ready model. The usefulness of precise spatiotemporal perturbation of genes extends beyond immunology. Optogenetic techniques have been widely adopted by neuroscience, light being an excellent signal to control individual neurons. The understanding of the genetical underpinnings of neural circuitry and regulation of subsequent events could be elucidated with the help of optogenetic CRISPR tools. Similarly, multicellular organisms develop in highly regulated spatiotemporal patterns, making developmental biology and regenerative medicine natural candidates for optogenetic applications. In conclusion, the findings presented here could facilitate the introduction of efficient optogenetic CRISPR gene editing *in vivo* for use in experimental animal models across scientific fields.

## Supplementary Material

gkaf213_Supplemental_File

## Data Availability

Illumina sequencing data for endogenous base editing are available at GEO accession number GSE275757.
