# Supplementary material for: Light-induced expression of gRNA allows for optogenetic gene editing of T lymphocytes in vivo
Source: Nucleic Acids Res. 2025 Mar 20;53(6):gkaf213. doi: 10.1093/nar/gkaf213 (PMC11925727; doi:10.1093/nar/gkaf213)
Supplement: gkaf213_Supplemental_File [file gkaf213_supplemental_file.pdf]

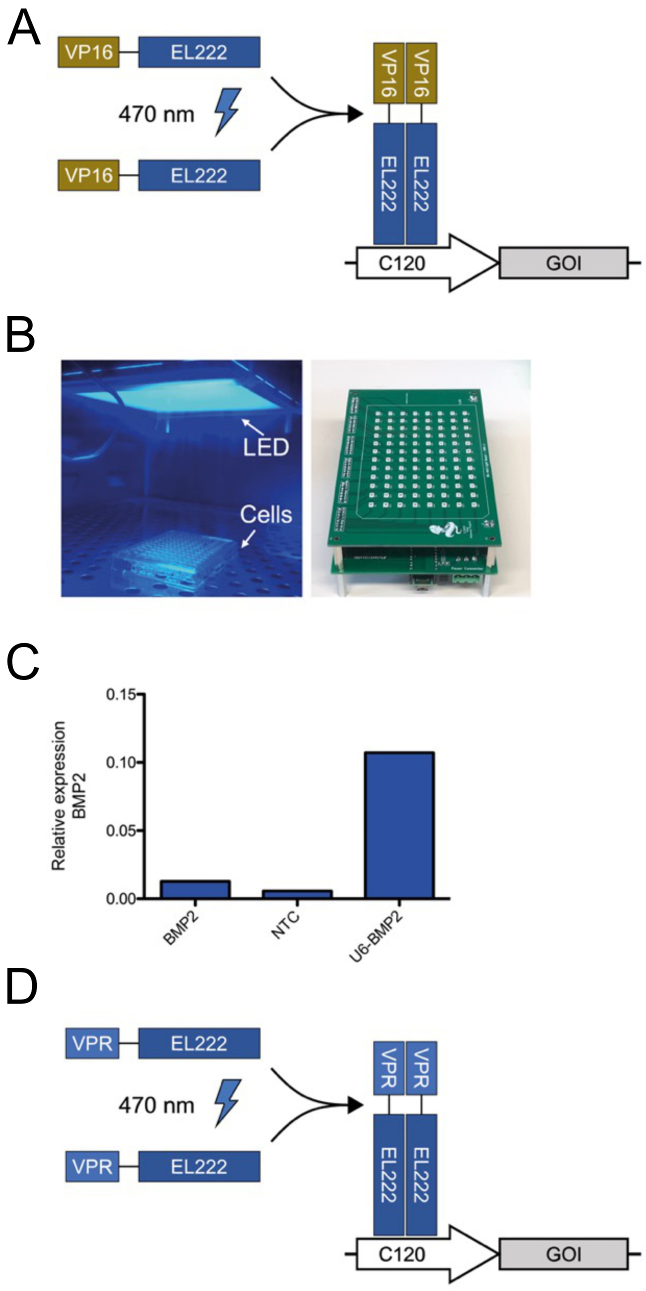

Supplementary Figure 1. VP16-EL222 and VPR-EL222 schematics and initial test
a, Schematics of the original VP16-EL222 system.
b, Transilluminator blue LED setups used in the optogenetic experiments. Overhead transilluminator on the left, 96 well transilluminator on the right.
c, VP16-EL222 based RGR production fails to activate significant expression of target endogenous gene with dCas9-VPR (U6-BMP2 is the same gRNA expressed constitutively from an hU6 promoter, positive control). Gene expression was normalized to *HPRT1* expression. The experiment was performed six times and one representative result is shown (mean of technical triplicates).
d, Schematics of the VPR-EL222 system.

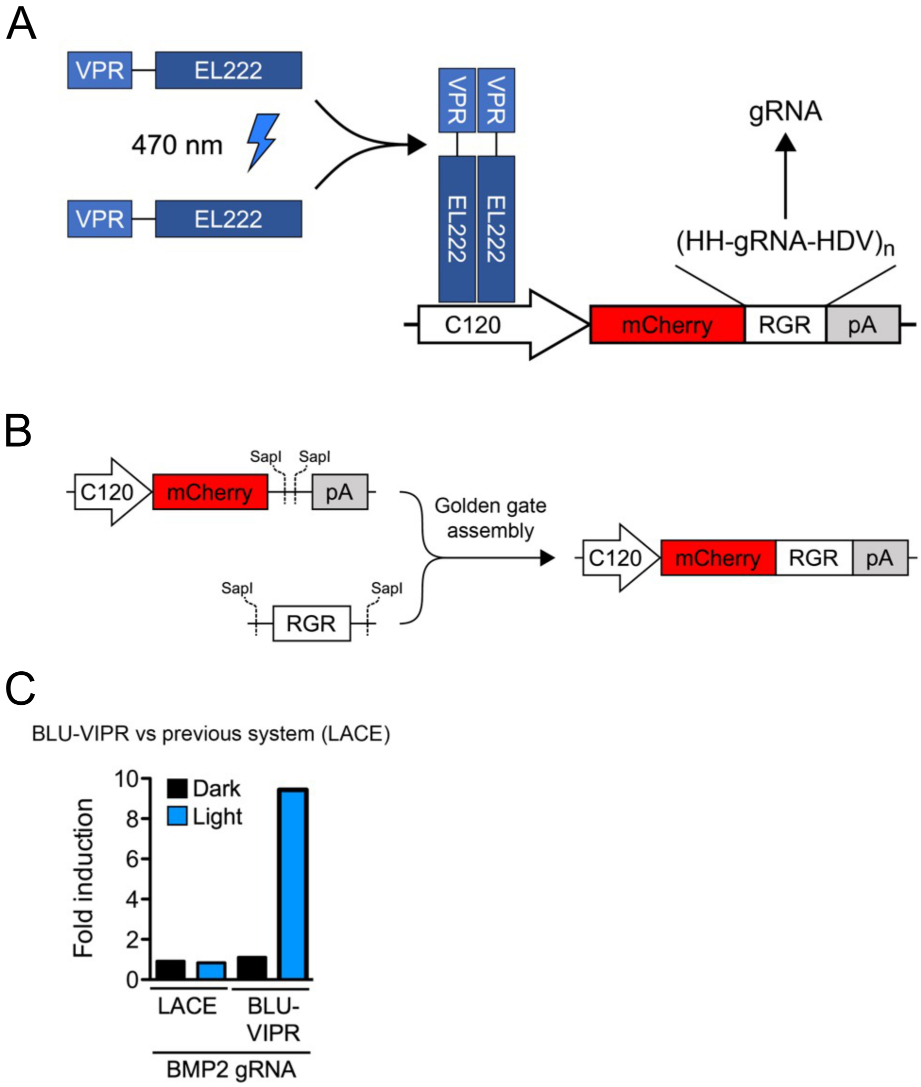

Supplementary Figure 2. BLU-VIPR system schematics and comparison to LACE
a, Schematics of the BLU-VIPR system.
b, Overview of cloning strategy for insertion of RGRs into BLU-VIPR plasmid. RGRs are designed with flanking SapI type IIS restriction enzyme sites, allowing for overhangs matching the vector backbone overhangs. A golden gate cloning, single step reaction results in the desired BLU-VIPR RGR plasmid.
c, BLU-VIPR outperforms previously reported optogenetic CRISPRa system (LACE) when a single gRNA is used to target the endogenous *BMP2* gene. The experiment was performed three times and one representative result is shown (mean of technical triplicates).

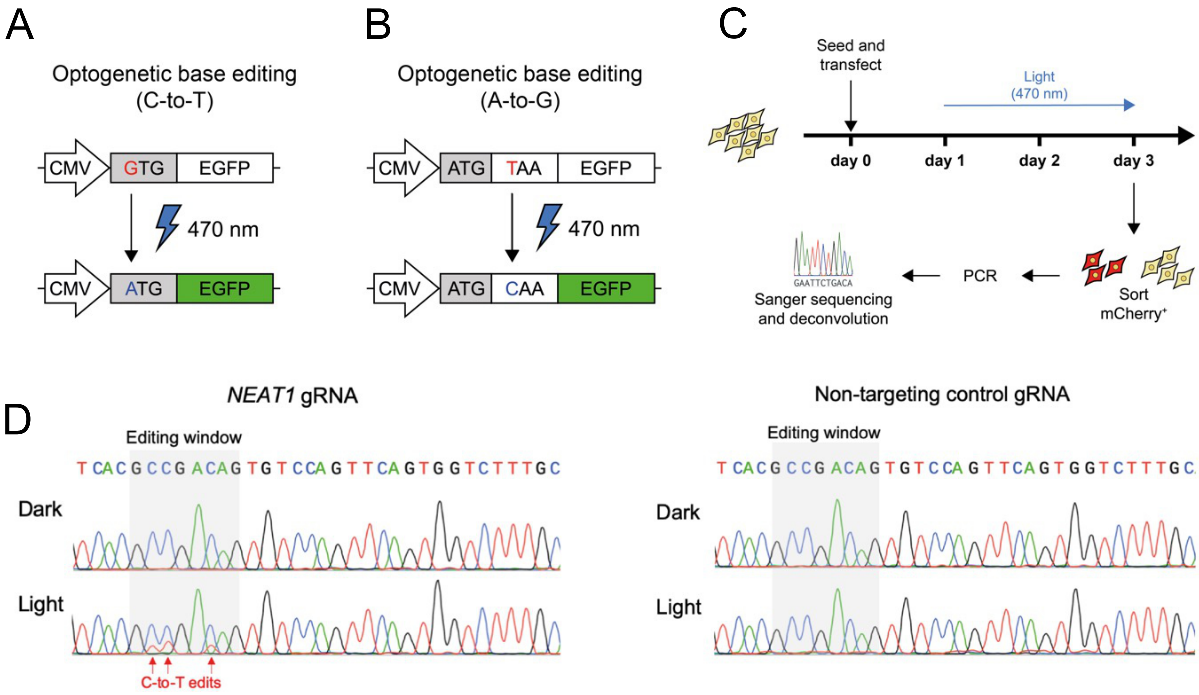

Supplementary Figure 3. Optogenetic base editing experimental setups

a, C-to-T base editing reporter cells turn EGFP positive after successful editing of C-to-T on the non-coding strand.

b, A-to-G base editing reporter cells turn EGFP positive after successful editing of A-to-G on the non-coding strand.

c, Overview of the optogenetic C-to-T base editing experiment targeting the endogenous lncRNA gene *NEAT1*.

d, validation of base editing using Sanger sequencing.

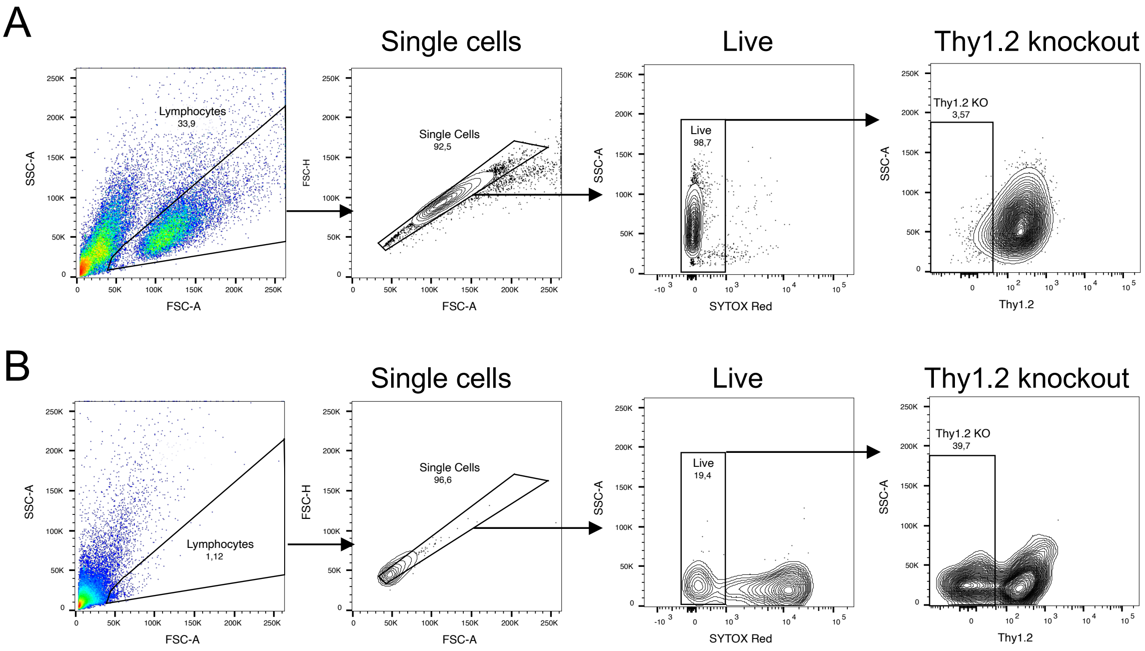

Supplementary Figure 4. Gating strategy for in vitro T lymphocyte experiments
a, Gating strategy for optogenetic CRISPR experiment in Cas9$^+$ Thy1.2$^+$ mouse T lymphocytes kept in the dark. T lymphocytes were gated for single cells and viability (SYTOX^{TM} Red negative) before being analyzed for Thy1.2 expression on the cell surface.
b, Gating strategy for optogenetic CRISPR experiment in Cas9$^+$ Thy1.2$^+$ mouse T lymphocytes illuminated with 470 nm light for 48 hours. T lymphocytes were gated for single cells and viability (SYTOX^{TM} Red negative) before being analyzed for Thy1.2 expression on the cell surface.

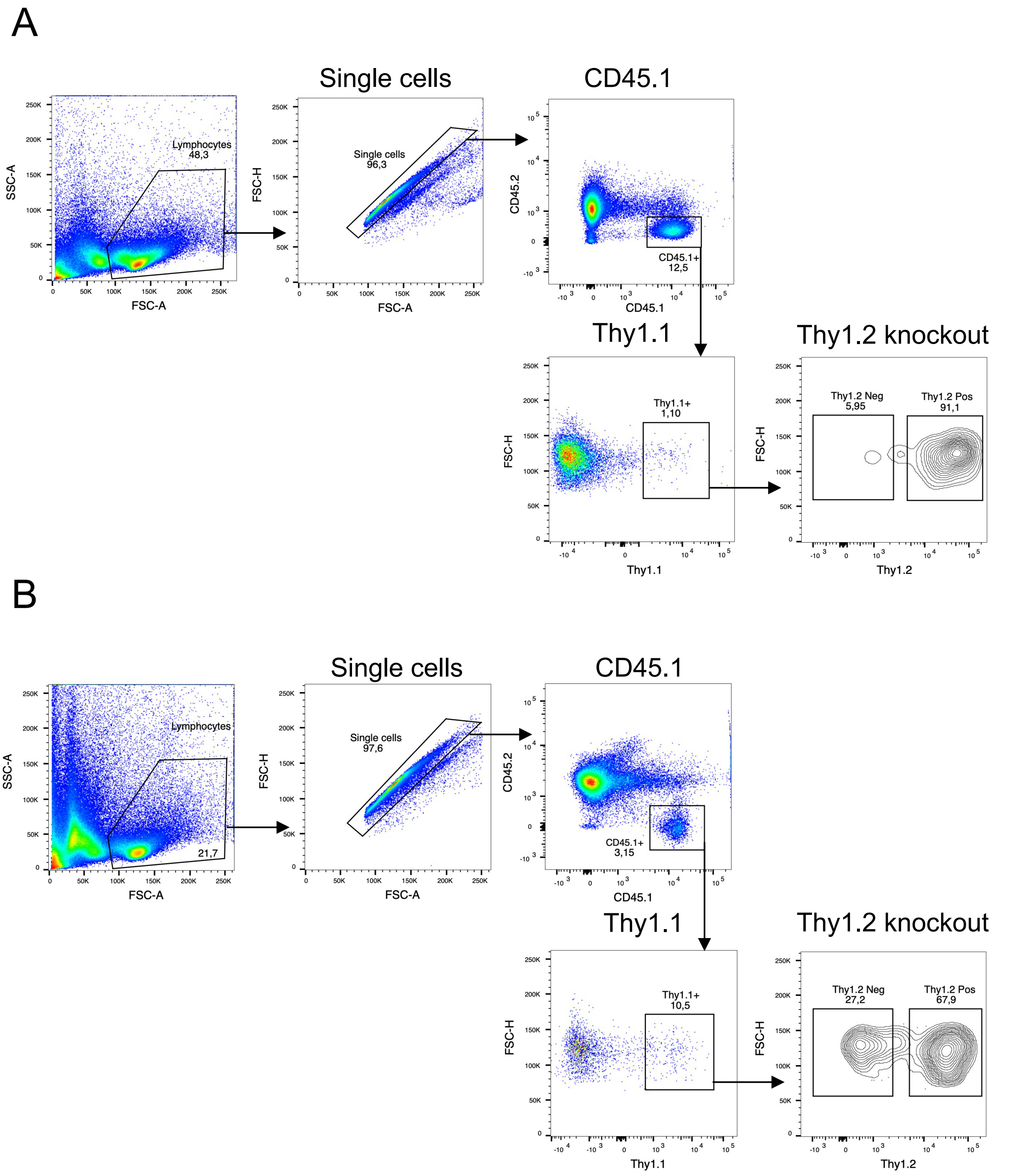

Supplementary Figure 5. Gating strategy for in vivo T lymphocyte optogenetic experiments
a, Gating strategy for in vivo optogenetic CRISPR experiment in Cas9^{+} Thy1.2^{+} mouse T lymphocytes kept in the dark.
b, Gating strategy for in vivo optogenetic CRISPR experiment in Cas9^{+} Thy1.2^{+} mouse T lymphocytes illuminated with 470 nm light for 1 hour.

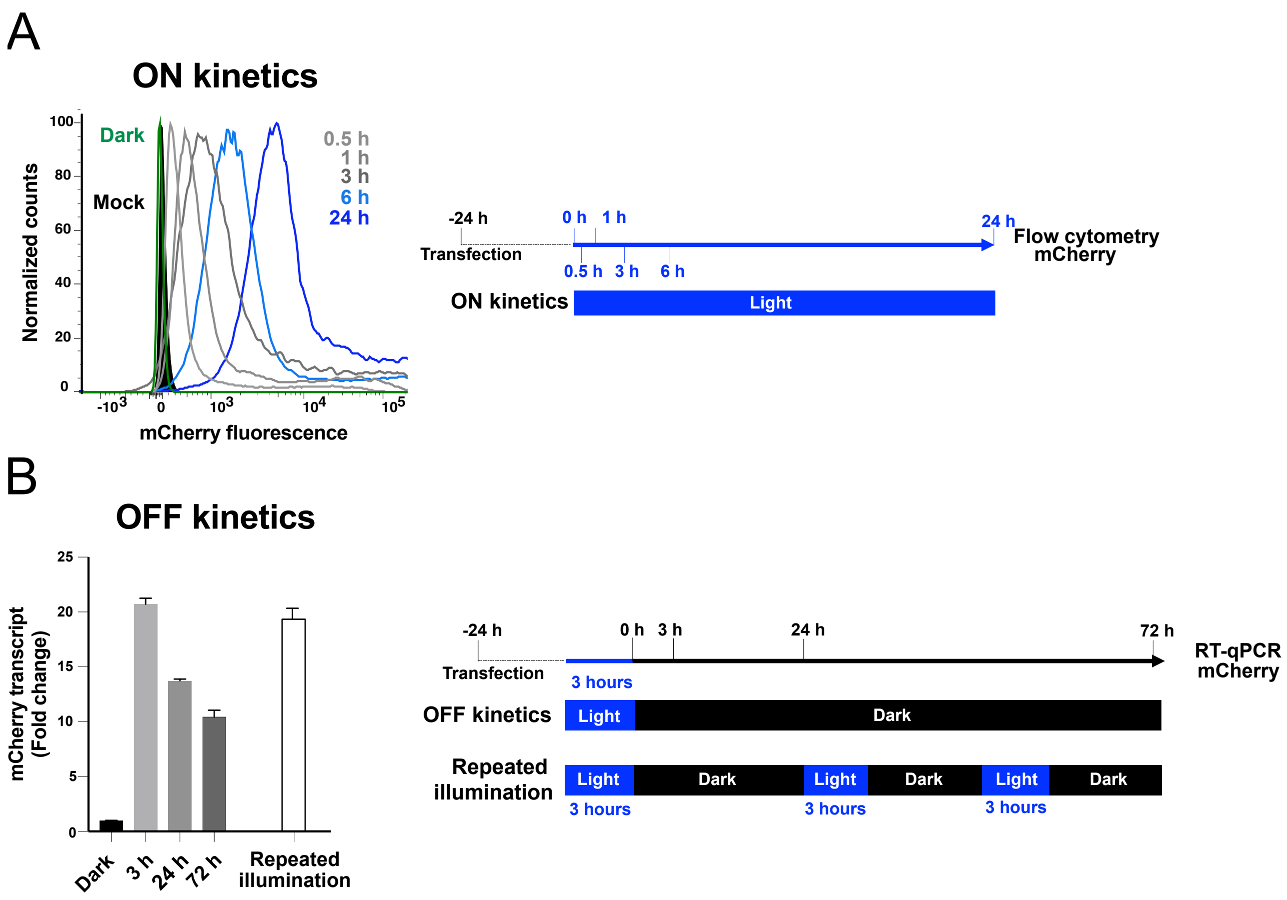

Supplementary Figure 6. ON/OFF kinetics of the BLU-VIPR system
a, For ON kinetics, HEK293T cells were transfected with BLU-VIPR plasmid and illuminated for 0 (Dark), 0.5, 1, 3, 6 or 24 hours. Cells were collected and mCherry fluorescence was quantified using flow cytometry.
b, For OFF kinetics, HEK293T cells were transfected with BLU-VIPR plasmid and illuminated for 3 hours. Cells were then collected at indicated time points and analyzed for mCherry transcripts. Repeated illumination was also performed to verify the potential of the system to reactivate

Supplementary Table 1
RGR elements and sequences used for cloning into BLU-VIPR vector. Green bases are SapI/LguI recognition sequences. Red bases in HH ribozyme sequence represent a variable region that must be the reverse complement of the first 6 bases immediately following the 3' end of the HH ribozyme (in blue).

Type IIs site	HH ribozyme	gRNA	HDV ribozyme	Type IIs site
TGTC<br>CCAC<br>**GCTC**<br>**TTC**T<br>TCC	**NNNNNN**CTG<br>ATGAGTCCG<br>TGAGGACGA<br>AACGAGTAA<br>GCTCGTC	**NNNNNN**NNNNNNNNNNNNNNG<br>TTTTAGAGCTAGAAATAGCAA<br>GTTAAAATAAGGCTAGTCCGT<br>TATCAACTTGAAAAAGTGGCA<br>CCGAGTCGGTGCTTTT	GGCCGGCATGGTCC<br>CAGCCTCCTCGCTG<br>GCGCCGGCTGGGCA<br>ACATGCTTCGGCAT<br>GGCGAATGGGAC	GTTT<br>**GAAG**<br>**AGC**G<br>CGCA<br>TGG

Supplementary Table 2.
List of all gRNA spacer sequences used in the optogenetic experiments.

**Target**	**Spacer**	**Cas**
NTC (non-targeting control)	GAACGACTAGTTAGGCGTGTA	SP-Cas9<br>LB-Cas12a
BMP2	GGCGAGCCGCGCCGCGAAGG	SP-Cas9
tdTomato (Cas9 nuclease Reporter)	GGGCCACTAGGGACAGGAT	SP-Cas9
PDGFB	TAAAGGAGAAGGGAGAGTGCGAG	LB-Cas12a
C-to-T Reporter	CACGGTCACCCTGACACGCT	SP-Cas9
A-to-G Reporter	CCTTATGACCCTGACACGCT	SP-Cas9
NEAT1	GCCGACAGTGTCCAGTTCAG	SP-Cas9
Thy1 (Thy1.2)	CGTGTGCTCGGGTATCCCAA	SP-Cas9
IL1RN	GGTACTCTCTGAGGTGCTC	SP-Cas9
HBG1/2	GCTAGGGATGAAGAATAAA	SP-Cas9

Supplementary Table 3
Primers used for RT-qPCR for the optogenetic CRISPRa experiments.

Target	Forward	Reverse
IL1RN	GGAATCCATGGAGGGAAGAT	TGTTCTCGCTCAGGTCAGTG
HBG1/2	GCTGAGTGAACTGCACTGTGA	GAATTCTTTGCCGAAATGGA
GAPDH	CAATGACCCCTTCATTGACC	TTGATTTTGGAGGGATCTCG

Supplementary Table 4
Primers used for genomic DNA PCR amplification of *NEAT1* gene sequence surrounding the targeting window for the optogenetic base editing experiments.

F	TAACTTACGGAGTCGCTCTACGGACTACCCCATCACAGAGTACTTTT
R	GGATGGGATTCTTTAGGTCCTGGGGTTTGTATGAACTTACTGGCATT
